# Lignocellulose degradation in *Protaetia brevitarsis* larvae digestive tract: refining on a tightly designed microbial fermentation production line

**DOI:** 10.1186/s40168-022-01291-2

**Published:** 2022-06-13

**Authors:** Kui Wang, Peiwen Gao, Lili Geng, Chunqin Liu, Jie Zhang, Changlong Shu

**Affiliations:** 1 State Key Laboratory for Biology of Plant Diseases and Insect Pests, Institute of Plant Protection, Chinese Academy of Agricultural Sciences, Beijing, 100193 China; 2 Hebei Key Laboratory of Soil Entomology, Cangzhou Academy of Agricultural and Forestry Sciences, Cangzhou, 061001 China

**Keywords:** Lignocellulose degradation, *Protaetia brevitarsis*, Transcriptome, Microbiome, Holobiont, CAZymes

## Abstract

**Background:**

The Scarabaeidae insect *Protaetia brevitarsis* (PB) has recently gained increasing research interest as a resource insect because its larvae can effectively convert decaying organic matter to plant growth-promoting frass with a high humic acid content and produce healthy, nutritional insect protein sources. Lignocellulose is the main component of PB larvae (PBL) feed, but PB genome annotation shows that PBL carbohydrate-active enzymes are not able to complete the lignocellulose degradation process. Thus, the mechanism by which PBL efficiently degrade lignocellulose is worthy of further study.

**Results:**

Herein, we used combined host genomic and gut metagenomic datasets to investigate the lignocellulose degradation activity of PBL, and a comprehensive reference catalog of gut microbial genes and host gut transcriptomic genes was first established. We characterized a gene repertoire comprising highly abundant and diversified lignocellulose-degrading enzymes and demonstrated that there was unique teamwork between PBL and their gut bacterial microbiota for efficient lignocellulose degradation. PBL selectively enriched lignocellulose-degrading microbial species, mainly from *Firmicutes* and *Bacteroidetes*, which are capable of producing a broad array of cellulases and hemicellulases, thus playing a major role in lignocellulosic biomass degradation. In addition, most of the lignocellulose degradation-related module sequences in the PBL microbiome were novel. PBL provide organic functional complementarity for lignocellulose degradation via their evolved strong mouthparts, alkaline midgut, and mild stable hindgut microenvironment to facilitate lignocellulosic biomass grinding, dissolving, and symbiotic microbial fermentation, respectively.

**Conclusions:**

This work shows that PBL are a promising model to study lignocellulose degradation, which can provide highly abundant novel enzymes and relevant lignocellulose-degrading bacterial strains for biotechnological biomass conversion industries. The unique teamwork between PBL and their gut symbiotic bacterial microbiota for efficient lignocellulose degradation will expand the knowledge of holobionts and open a new beginning in the theory of holobionts.

Video Abstract

**Supplementary Information:**

The online version contains supplementary material available at 10.1186/s40168-022-01291-2.

## Background

Animal domestical and agriculture play an essential role in food supply, especially in the conversion of plant biomass to proteins, ranging from dairy products to beef, poultry, fish, eggs, and pork [1]. Currently, great pressures imposed by the global population growth have not only increased the scale of the livestock industry but have also facilitated a search for novel, sustainable protein sources. Domestic insects that convert agricultural waste to edible proteins have been acknowledged as a feasible strategy due to their broad range of feed sources and high feed conversion rates [2, 3].

*Protaetia brevitarsis* (PB) (Lewis 1879; NCBI: txid348688; Coleoptera: Scarabaeidae: Cetoniinae; homotypic synonym: *Cetonia brevitarsis*, *Liocola brevitarsis*, *Pachnotosia brevitarsis*, *Potosia* (Liocola) *brevitarsis*, *Potosia brevitarsis* (Lewis, 1879)) is a promising candidate organism [4]. PB larvae (PBL) are saprophagous and can feed on a large variety of organic matter, from decaying plant residues to the humus, livestock waste, and spent mushroom substrates. Based on the special feeding habits of PBL and the amount of collectible organic matter, the feed sources available to feed PBL are extensive. Furthermore, PBL can effectively digest plant residues and accumulate proteins and lipids for the larval development. Dried mature PBL contain 54.16–67.07% protein, 9.91–19.38% lipids, and a wide variety of micronutrients [4]. In addition, our recent studies have demonstrated that PBL can effectively convert plant residues to nonphytotoxic frass fertilizers with a high humic acid content [5, 6]. These reports indicate that lignocellulosic biomass can be efficiently digested in the PBL digestive tract.

In nature, saprophagous scarab larvae, including PBL, are important litter transformers and play an important role in the terrestrial carbon cycle [5, 7]. They have evolved a highly compartmentalized digestive tract that helps them obtain nutrients and energy from lignocellulose [8, 9]. The typical alimentary tract of scarab larvae is divided into the following three major sections: a foregut used for food storage, a long midgut occupying most of the length of the body cavity, and a modified expanded hindgut, which is often referred to as a fermentation chamber [10]. Like termites, scarab larvae possess a highly alkaline midgut, which is believed to help increase the solubility of organic polymers, thus rendering the organic components accessible for digestion in subsequent less-alkaline compartments [8, 10]. These larvae also usually possess an enlarged hindgut with a near-neutral environment harboring a dense and diverse microbial community, analogous to that in the microorganism-rich rumen of higher mammals, which is the primary site of microbial fermentation for lignocellulose digestion [10].

Lignocellulose is mainly composed of lignin, cellulose, and hemicellulose, forming a highly complex and varying polymeric structure that is highly recalcitrant to degradation and thus requires a consortium of carbohydrate-active enzymes (CAZymes) that act in synergism to provide its complete decomposition [11]. Lignin degradation is an enzymatic oxidation catalyzed by two main groups of enzymes, namely, lignin-modifying enzymes (LMEs) and lignin-degrading auxiliary (LDA) enzymes [12]. In contrast to lignin degradation, enzymatic degradation of cellulose and hemicellulose is mainly mediated by a process of hydrolysis through the action of glycoside hydrolases. Commonly, the process of cellulose degradation involves a set of three enzymes, endoglucanase, exoglucanase, and β-glucosidase, while the depolymerization of hemicellulose requires endo-hemicellulases, exo-hemicellulases, and debranching enzymes that cleave side chains of the polymers or associated oligosaccharides [11, 13]. In addition, recent studies have indicated that the efficiency of lignocellulose degradation can be remarkably improved by the cooperative action of lytic polysaccharide monooxygenases (LPMOs), which are able to directly oxidize and depolymerize insoluble crystalline substrate surfaces or soluble hemicellulosic substrates such as xyloglucan, xylan, and β-glucans [14–16]. In natural ecosystems, the degradation of lignocellulosic biomass is mainly dependent on a repertoire of enzymes produced by bacteria and fungi [11, 12]. However, more efficient degradation can be achieved by host eukaryotes working together with their gut microorganisms (often referred to as a “holobiont”) [17–19]. Recent data have suggested that isopod holobionts are promising models for lignocellulose degradation and that terrestrial isopods usually obtain complementarity benefits from their microbiota [19]. Compared with other lignocellulose decomposers, such as the termite gut [20, 21], earthworm gut [22], or cattle rumen [23], PBL possess more abundant gut microbial communities [24], which may indicate the availability of abundant and novel lignocellulosic enzymes or microbial candidates in the PBL gut ecosystem. In the present work, we combined genomic, transcriptomic, and metagenomic approaches at the holobiont level for the first time to provide new insights into the mechanisms underlying highly efficient lignocellulose degradation by PBL. The investigation included (i) identifying lignocellulose-degrading CAZymes and lignocellulose-binding modules present in both the host and microbiota, (ii) characterizing microbial taxa that contribute lignocellulose degradation-related genes, and (iii) recovering individual lignocellulolytic species from the microbiota through a metagenomic binning approach. This investigation improves our understanding of the lignocellulose degradation mechanisms in PBL and also contributes to applications in edible insect farming as well as in biofuel and biomaterial production.

## Methods

### Preparation of samples

The PB laboratory population was derived from a field population collected in Gongzhuling, Jilin Province, China [25], and reared in a constant environment in an incubator at 26°C, 40–60% relative humidity, and a photoperiod of 12-h light to 12 h dark. The larvae were fed corn straw, which was crushed into approximately 1-cm pieces with 50% moisture content. Third-instar larvae were selected and chilled on ice for dissection. After surface sterilization using 70% ethanol, the midgut and hindgut were dissected for subsequent analysis.

### PBL gut transcriptome analysis

To prepare midgut and hindgut tissues, the dissected midgut and hindgut were washed in a cold 125-mM NaCl solution after removing gut contents. Subsequently, the washed gut tissue was transferred into a homogenizer and homogenized with TRIzol reagent (Invitrogen, USA), and the RNA was extracted according to the manufacturer’s protocol. The RNA quality and quantity were determined with gel electrophoresis and a NanoDrop spectrophotometer (Thermo Fisher, USA). Then, RNA sequencing libraries were generated using an Illumina TruSeq-stranded mRNA Library Prep Kit (Illumina, USA), and sequencing was performed on an Illumina HiSeq 2500 sequencer (Illumina, USA) to produce 2 × 150 bp paired-end reads. When the raw reads were produced, quality control, adapter trimming, and quality filtering were performed by Fastp (version 0.21.0) [26]. Finally, clean reads were deposited in the NCBI Sequence Read Archive (SRA). The SRA accessions SRR5038971, SRR5039436, SRR5039445, and SRR14128221, SRR14132028, and SRR14132050 correspond to midgut and hindgut samples from three larvae, respectively (Additional file 1: Table S1).

To determine the transcriptomic expression profiles in the PBL gut, the clean reads were aligned to the PB reference genome [25] using Spliced Transcripts Alignment to a Reference (STAR, version 2.7.8a) [27], and the resulted read alignment was sorted using SAMtools (version 1.15.1) [28]. Subsequently, the expression of genes was analyzed using StringTIE (version 2.1.5) [29] in terms of the fragments per kilobase of transcript per million mapped read (FPKM) values of encoding genes. Each gene expression level was normalized to its length for each replicate using the FPKM method which eliminates the influence of varying gene lengths and sequencing discrepancies in the calculation of gene expression. The paired Student’s *t* test was used to evaluate the significance of the differences between the FPKM values of the midgut and hindgut sample groups.

### Gut metagenome sequencing and assembly

To prepare enough DNA for gut metagenomic sequencing, ten 3rd-instar PBL fed with corn straw were dissected, and the midgut and hindgut contents were pooled together. The DNA of the pooled midgut or hindgut contents was extracted using an Axyprep Multisource Genomic DNA Miniprep Kit (AxyGen, USA).

Then, paired-end TruSeq DNA PCR-Free libraries with insert sizes of 250 and 420 bp were constructed from the samples, and an Illumina HiSeq 2500 sequencer (Illumina, USA) was used to sequence the libraries to produce 2 × 150 bp paired-end reads. The quality of raw reads was checked with FastQC (version 0.11.9, https://www.bioinformatics.babraham.ac.uk/projects/fastqc/). Then, the adapters were trimmed using Trimmomatic (version 0.39) [30]. Reads shorter than 36 bp were removed. The clean reads were deposited to the SRA, where the SRA accessions SRR14139157-SRR14179698 were midgut samples and SRR14150473-SRR14209386 were hindgut samples (Additional file 1: Table S1). To assist genome binning, available PBL hindgut (AHG, BHG, and MHG) and frass (AFR, BFR, and MFR) metagenomes in the SRA database were also employed (Additional file 1: Table S1).

High-quality reads from midgut and hindgut samples were pooled, and the metagenomic classifier MetaPhlAn (version 3.0.13) [31] was employed for profiling all the reads in the community with default parameters to infer the taxonomic composition of the microbial community. Subsequently, MEGAHIT (version 1.2.9) [32] was used for coassembly with default parameters. Following assembly, reads were mapped to assembled contigs using the BWA-MEM algorithm [33] and SAMtools (version 1.6) [28] to obtain coverage information. Contig binning was conducted to recover individual genomes based on both tetranucleotide frequencies and sequence coverages. MaxBin 2.0 (version 2.2.7) [34] and MetaBAT 2 (version 2.12.1) [35] were used for independent binning using contigs longer than 1500 bp and clean reads from each sample. All generated bins were aggregated and then dereplicated using dREP (version 1.4.3) [36] with default parameters. CheckM (version 1.0.7) [37] was used to estimate the genome completeness and contamination of all dereplicated bins. The binned genomes were assigned to taxa following the procedure proposed by Stewart et al. [38] using the MAGpy [39] program. The phylogenetic tree of bins was built based on a concatenated protein sequence alignment using PhyloPhlAn (version 3.0.60) [40] and was annotated through iTOL (version 5, https://itol.embl.de) [41]. The relative abundance of individual taxa was measured by mapping the clean reads against binned scaffolds after a normalization step based on the size of the relevant genome bins.

### Gene functional annotation

Before functional annotation, gene prediction of metagenomic contigs or bins was performed. Protein-coding sequences (CDSs) from coassembled metagenomic contigs were predicted using Prodigal (version 2.6.3) with the option –p meta [42]. The CDSs, rRNAs, and tRNAs of each metagenomic bin were predicted using the Prokka (version 1.13) [43] included in the metaWRAP [44] pipeline, with default parameters.

In the present work, gene functional annotation was focused on the lignocellulose degradation process. The Carbohydrate-Active enZYmes (CAZy) modules were identified using the CAZy database [45]. dbCAN2 was employed for annotating CAZy families from the PBL genome, transcriptome, metagenome, and individual metagenomic bins through the HMMER search approach, with an E-value threshold of 0.0001 [46]. CAZy families include auxiliary activities (AAs), carbohydrate esterases (CEs), glycoside hydrolases (GHs), glycosyltransferases (GTs), polysaccharide lyases (PLs), and carbohydrate-binding modules (CBMs). Multiple CAZy families present in a single sequence were allowed. All protein sequences identified as CAZy modules were then imported into Hotpep [47] to predict their enzymatic activity and to confirm their implication in lignocellulose degradation.

To assign the microbial sources of lignocellulose degradation-related genes, all protein sequences identified as lignocellulose-degrading CAZymes and lignocellulose-binding modules were searched against the NCBI non-redundant (NR) protein database (ftp://ftp.ncbi.nlm.nih.gov/blast/db/, March 2021) using DIAMOND (version 0.9.24.125) [48] with an E-value cutoff of 0.0001. Then, the DIAMOND outputs were imported into MEGAN (version 6.21.5) [49] for taxonomic assignment on the basis of the lowest common ancestor (LCA) algorithm. The Wilcoxon test was employed to compare the sequence identity differences of lignocellulose degradation-related proteins between the midgut and hindgut microbiome sample groups.

## Results

### General features of the PBL gut transcriptome and metagenome

To understand the lignocellulose degradation mechanism at the holobiont level, PBL gene transcripts in the gut were determined by transcriptome sequencing. After adapter trimming and quality filtering, a total of 104,357,624 and 134,887,484 high-quality clean reads, encompassing 13,044,703,000 and 20,171,214,779 bp of sequences, were generated from midgut and hindgut libraries, respectively. After alignment to the PB reference genome, a total of 8505 genes were identified to be expressed in the midgut and hindgut (Table 1). Among these, 8391 genes were expressed in the midgut, 8159 genes were expressed in the hindgut, and 8045 of them were expressed in both the midgut and hindgut (FPKM > 0, Additional file 2: Table S2).

**Table 1:** Data summary of PBL transcriptome and metagenome

Sample	Host			Microbiome	
	Genome^a^	Midgut transcriptome	Hindgut transcriptome	Midgut	Hindgut
Total reads^b^	-	104,357,624	134,887,484	1,643,212,636	1,985,361,688
Total bases^b^	-	13,044,703,000	20,171,214,779	165,332,536,581	199,652,618,725
Contigs	-	-	-	1,337,306	3,930,676
N50	-	-	-	1792	1854
CDS	33,654	8,391	8,159	2,184,816	6,438,643
CAZy modules	700	149	142	40,338	125,682
LDMs	182	40	40	12,845	40,616
Cellulases	2	1	1	280	1031
Hemicellulases	73	14	14	6516	18,781
Hemicellulases and cellulases	31	15	15	2493	7741
Ligninases	42	3	3	1038	2089
Lignocellulose-binding modules	34	7	7	2518	10,974

*LDMs* lignocellulose degradation-related modules

^a^PB genome data from Wang et al. (2019)

^b^Reads and bases after quality trimming

For PBL gut metagenome sequencing, we obtained 1,643,212,636 and 1,985,361,688 high-quality clean reads from midgut and hindgut samples, respectively, encompassing 165,332,536,581 base pairs (bp) and 199,652,618,725 bp of sequences. These were assembled into 1,337,306 contigs coding for 2,184,816 genes in the midgut and 3,930,676 contigs coding for 6,438,643 genes in the hindgut (Table 1).

### Taxonomic composition of the PBL gut microbiota

**Fig. 1:**
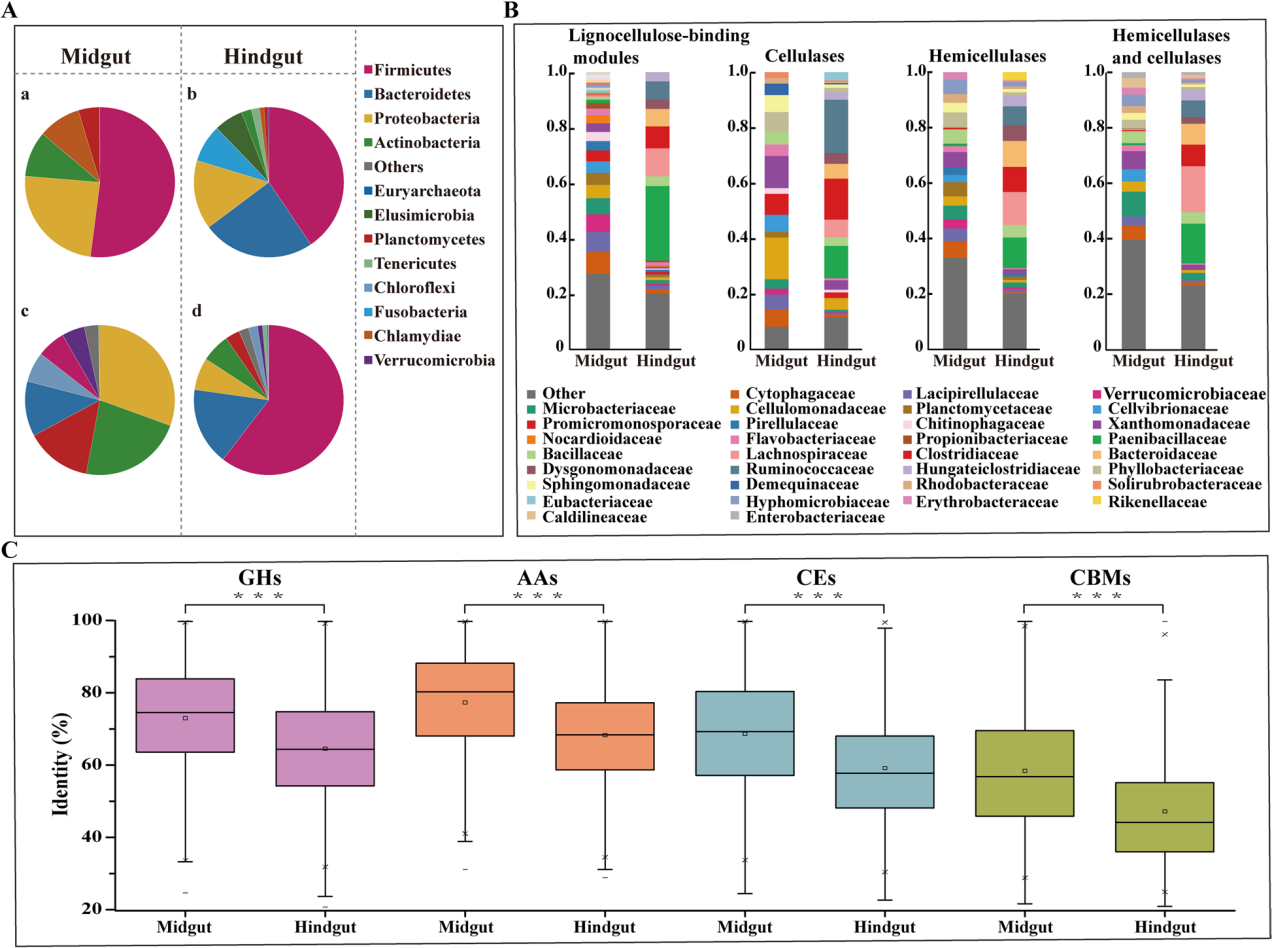
Taxonomic composition at the phylum level by the relative abundances of metagenomic reads in the midgut (**A-a**) and hindgut (**A-b**) or by the lignocellulose degradation-related modules predicted in the midgut (**A-c**) and hindgut (**A-d**) microbiome. Family level taxonomic distribution of the lignocellulose degradation-related modules in the midgut and hindgut microbiome (**B**). Similarity distribution between lignocellulose degradation-related proteins (GHs, AAs, CEs, and CBMs) and the best hit in the NCBI NR protein database (**C**). Box plots show the percentage sequence identity of lignocellulose degradation-related proteins encoded in the hindgut were more novel than those in the midgut, and asterisks (***) indicate statistically significant differences (*p* < 0.001, Wilcoxon test)

To examine whether a unique lignocellulose-degrading microbial community was enriched in the PBL gut, taxonomic distribution based on the reads from midgut and hindgut metagenome samples was analyzed at the species level (Additional file 3: Table S3). Consistent with our previous analysis based on 16S rRNA gene pyrosequencing [24], the microbial composition of the PBL midgut and hindgut was similar, but the relative abundance was different. Our taxonomic profiling analysis indicated that the microbial communities of the midgut and hindgut were composed of six and ten phyla, respectively (> 0.1% abundance). The phyla *Firmicutes*, *Bacteroidetes*, and *Proteobacteria* were the predominant bacteria in the hindgut, accounting for approximately 80% of the hindgut microbial communities. In contrast, *Firmicutes* and *Proteobacteria* were the most abundant phyla in the midgut, accounting for 76.31% of the midgut microbial communities found in this work. In addition to the above phyla, *Fusobacteria* (8.14%) and *Elusimicrobia* (6.25%) were also abundant in the hindgut but were not detected in the midgut microbial communities (Fig. 1A). At the family level, 26 and 23 families (> 0.1% abundance) were detected in the midgut and hindgut microbial communities, respectively (Additional file 3: Table S3). Bacillaceae from the phylum *Firmicutes* was the most abundant family in both the midgut and hindgut, accounting for 50.44% and 33.24% of the microbial communities, respectively. Bacteroidaceae from the phylum *Bacteroidetes* was abundant in the hindgut, accounting for 23.25% of the microbial communities; however, it represented only 0.01% in the midgut. These data suggested that many bacterial species were enriched in the hindgut.

### Characterization of CAZy modules in the host and PBL gut microbiome

To identify CAZy modules from the PBL holobiont, the coding genes from the PB genome and PBL gut transcriptome and metagenome were screened against the CAZy database (http://www.cazy.org). The results indicated that a total of 344 CAZy families were identified in the PBL holobiont (Additional file 4: Table S4). For the host, a total of 700 CAZy modules from 89 CAZy families were identified in the genome, and 149 CAZy modules from 58 CAZy families were confirmed to be expressed in the gut, where 149 modules were expressed in the midgut and 142 were expressed in the hindgut (FPKM > 0, Additional file 5: Table S5). Among these modules, 45 modules were expressed at a significantly higher level in the midgut than in the hindgut, while 17 were expressed at a significantly higher level in the hindgut than in the midgut (*p* < 0.05, Additional file 5: Table S5). For the PBL gut microbiome, a total of 166,020 CAZy modules from 343 CAZy families were identified, including 40,338 CAZy modules from the midgut microbiome and 125,682 CAZy modules from the hindgut microbiome, accounting for 1.76% and 1.75% of the total genes in the midgut and hindgut gene catalogs, respectively.

Among these CAZy modules, GTs catalyze the formation of glycosidic linkages to form glycosides and are essential for biological development and environmental adaptation. However, in the PBL holobiont, GT was not the largest family but instead constituted the second largest family. There were 95 different GT families, representing 83.33% of all known GT families in the CAZy database. A total of 44,610 GT modules were identified, including 44,453 modules and 157 modules in the microbiome and host genome, respectively. GT2 was the most prominent CAZy family in the PBL holobiont, with 15,745 modules representing 9.48% of microbiome CAZy modules and 8 modules representing 1.14% of host CAZy modules.

GHs, CEs, PLs, and AAs catalyze the breakdown or modification of carbohydrates and glycoconjugates, which are important for the hydrolysis and utilization of lignocellulose. In the PBL holobiont, GHs were the dominant class and comprised 124 different families, representing approximately 72.51% of all known GH families in the CAZy database. For the gut microbiome, a total of 52,405 GH modules were identified, including 12,565 modules in the midgut and 39,840 modules in the hindgut. In contrast, only 129 GH modules were identified in the PB genome, among which 52 were expressed in both the midgut and hindgut. In addition, 16 CE families with 19,206 modules in the microbiome and 102 modules in the host, 23 PL families with 3224 modules in the microbiome and one in the host, and 10 AA families with 3267 modules in the microbiome and 42 modules in the host were also identified.

CAZymes often display a modular structure with noncatalytic modules, i.e., CBMs, appended to the adjacent enzymatic modules. In the PBL holobiont, we identified 73 CBM families with 26,662 modules in the microbiome and 269 modules in the host. In addition to CBMs, 15,731 S-layer homology domains (SLHs) and 1072 cellulosome-binding domains (548 cohesins and 524 dockerins) were also detected and identified as docking modules in the PBL gut microbiome. The presence of these modules suggested the potential for active cellulosome-mediated lignocellulose plant cell wall degradation in the PBL gut.

### Lignocellulose degradation-related CAZy modules in the PBL holobiont

**Fig. 2:**
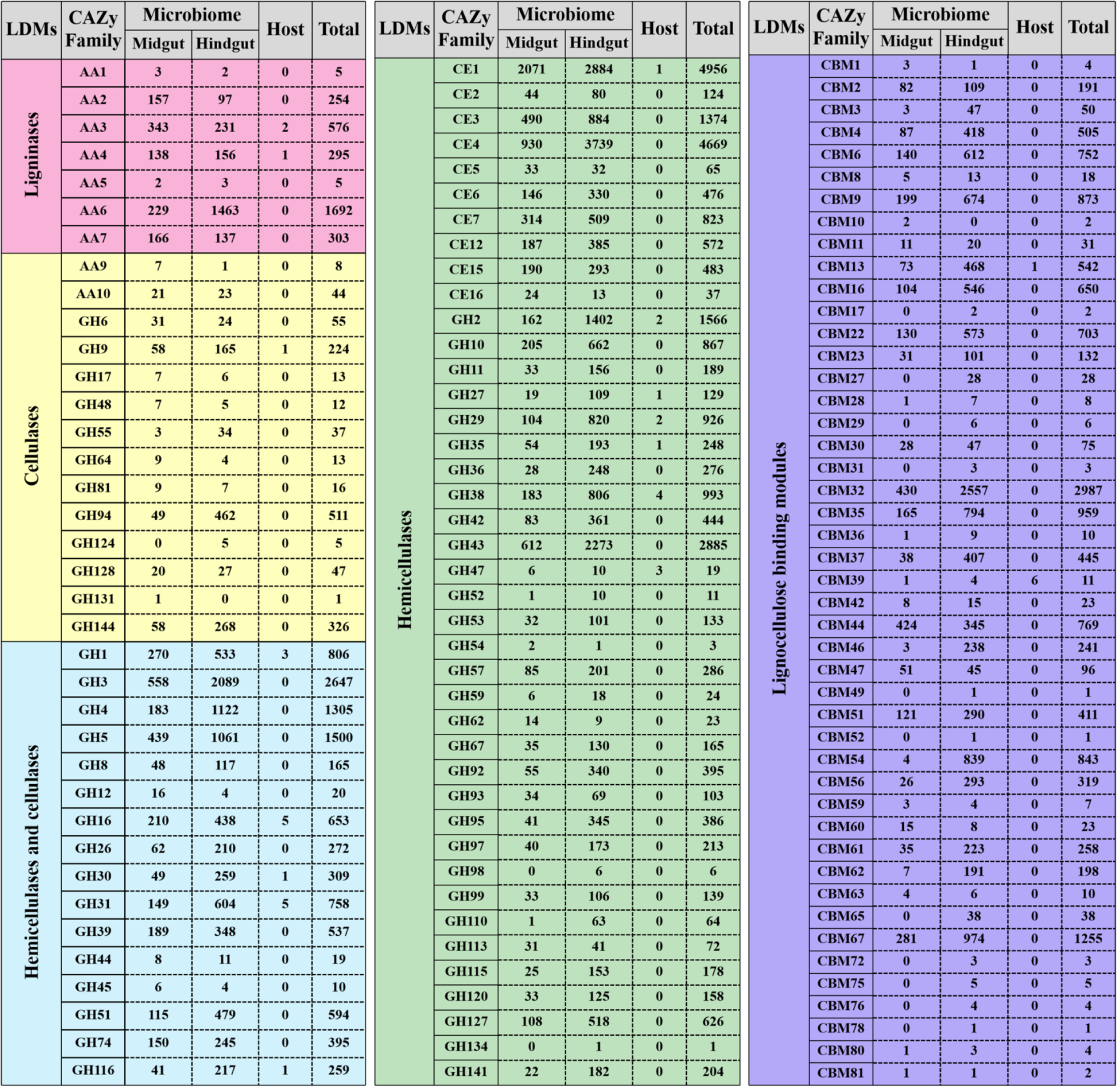
Distribution of lignocellulose-degrading CAZymes and lignocellulose-binding modules in the PBL gut gene catalog. Presented are the total numbers of CAZy modules for each family in the host gut transcriptome and in the gut metagenome

Then, we focused on the CAZymes known as lignocellulose-degrading enzymes (cellulases, hemicellulases, and ligninases) and CBMs known as lignocellulose-binding modules among all identified CAZy families in the PBL holobiont (Fig. 2; Table 1; Additional file 6: Table S6). In total, 40,117 lignocellulose-degrading CAZymes were identified in the PBL holobiont, including 39,969 modules in the microbiome and 148 modules in the host (33 in the transcriptome) (Additional file 7). These modules were from 78 lignocellulose-degrading CAZy families composed of 59 GH families, ten CE families, and nine AA families. Additionally, 13,526 lignocellulose-binding modules from 46 CBM families were found in the PBL holobiont, including 13,492 modules in the microbiome and 34 modules in the host (seven in the transcriptome). The hindgut microbiome contained most of the lignocellulose-degrading CAZymes and lignocellulose-binding modules, representing 74.10% (*N*=29,642) and 81.34% (*N*=10,974), respectively.

**Fig. 3:**
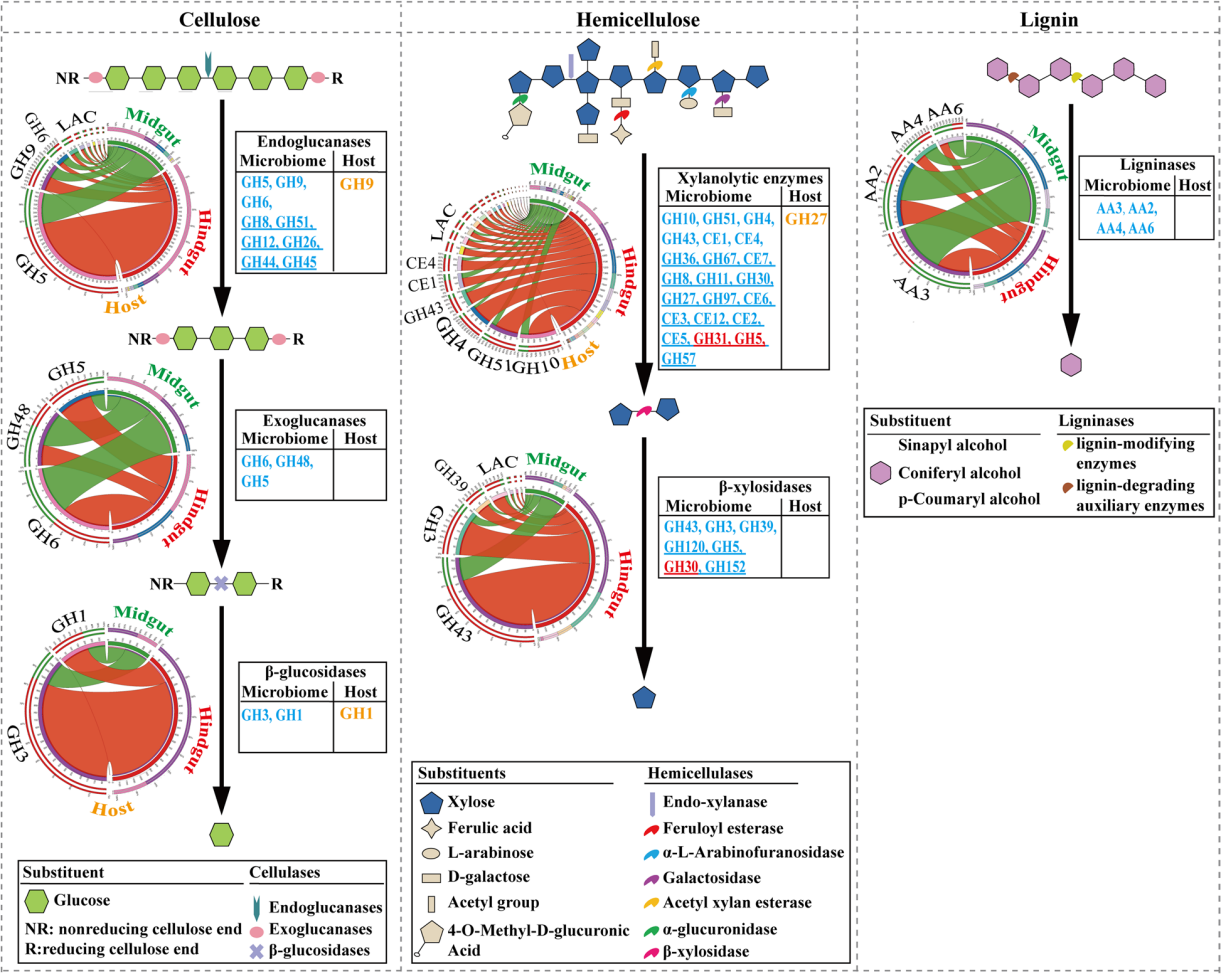
Cooperative model of cellulases, hemicellulases, and ligninases in lignocellulose degradation in the PBL holobiont. Diagrams represent the CAZy families contributed by the host (transcriptome, orange), the midgut microbiome (green), and the hindgut microbiome (red). The CAZy families present in both the midgut microbiome and hindgut microbiome were shown in blue. The underline represents the CAZy families with low abundance (LAC)

The modification and degradation of lignin have been identified as an essential step for efficient lignocellulosic biomass deconstruction [50], and lignin consumption is mainly accomplished by LMEs and LDA enzymes. LMEs are classified as laccases (EC 1.10.3.2; AA1), manganese-dependent peroxidases (EC 1.11.1.13; AA2), lignin peroxidases (EC 1.11.1.14; AA2), and versatile peroxidases (EC 1.11.1.16; AA2). Although LDA enzymes are unable to degrade lignin on their own, the lignin degradation process can be further enhanced by the action of these enzymes. In the present work, seven AA families (AA1, AA2, AA3, AA4, AA5, AA6, and AA7) involved in lignin modification and degradation were identified in the PBL holobiont, including 1038 modules in the midgut microbiome and 2089 modules in the hindgut microbiome, as well as 42 modules in the host (three in the transcriptome) (Fig. 2; Additional file 6: Table S6). Furthermore, enzymatic activities were functionally predicted by Hotpep, and the data showed that abundant peroxidases (AA2, EC 1.11.1.13, EC 1.11.1.14) were functionally identified in the microbiome, including 80 modules in the midgut and 53 modules in the hindgut. Additionally, four types of LDA enzymes were also functionally identified in the microbiome, including aryl-alcohol oxidases (44 modules; AA3, EC 1.1.3.7), cellobiose dehydrogenases (130 modules; AA3, EC 1.1.99.18), vanillyl-alcohol oxidases (54 modules; AA4, EC 1.1.3.38), and p-benzoquinone reductases (51 modules; AA6, EC 1.6.5.6) (Fig. 3; Additional file 8: Table S7). Notably, no laccase was functionally identified in either the PBL host or the gut microbiome, and neither LMEs nor LDA enzymes were identified in the PBL gut transcriptomes. Following the partial modification and degradation of lignins, celluloses, and hemicelluloses present in the plant biomass are released and can be attacked by a series of enzymes.

Cellulose is a linear polymer of β-d-glucose in which glucose units are linked together by β-1,4-glycosidic bonds. The depolymerization process of celluloses is as follows: first, endoglucanases (EC 3.2.1.4) randomly attack cellulose fibrils, which reveals sites for subsequent attack by exoglucanases; then, exoglucanases (EC 3.2.1.91 and EC 3.2.1.176), also known as cellobiohydrolases, remove monomers and dimers from the reducing/nonreducing ends of the glucan chain; and finally, β-glucosidases (EC 3.2.1.21) hydrolyze glucose dimers and, in some cases, cellulose-oligosaccharides to glucose [11]. In this work, a total of 30 CAZy families known to exhibit cellulase activity were identified in the PBL holobiont, corresponding to 11,578 modules, including 11,545 modules in the microbiome and 33 modules in the host (16 in the transcriptome). Among the 30 CAZy families, sixteen GH families (GH1, GH3, GH4, GH5, GH8, GH12, GH16, GH26, GH30, GH31, GH39, GH44, GH45, GH51, GH74, and GH116) contained both hemicellulases and cellulases; fourteen CAZy families, including two AA families (AA9 and AA10) and twelve GH families (GH6, GH9, GH17, GH48, GH55, GH64, GH81, GH94, GH124, GH128, GH131, and GH144), contained only cellulases. They were all present in the microbiome, while only six GH families (GH1, GH9, GH16, GH30, GH31, and GH116) were present in the gut transcriptome (Fig. 2; Additional file 6: Table S6). Among them, nine GH families (GH5, GH6, GH8, GH9, GH12, GH26, GH44, GH45, and GH51) were functionally identified as endoglucanases (EC 3.2.1.4), corresponding to 168 modules in the midgut microbiome and 349 modules in the hindgut microbiome, and only one module (GH9) was present in the gut transcriptome. Two GH families (GH1 and GH3) were functionally identified as β-glucosidases (EC 3.2.1.21), corresponding to 313 modules in the midgut microbiome and 1173 modules in the hindgut microbiome, and only two GH1 genes were present in the gut transcriptome. Three GH families (GH5, GH6, and GH48) were functionally identified as cellobiohydrolases (EC 3.2.1.91 and EC 3.2.1.176), corresponding to 14 modules in the midgut microbiome and 15 modules in the hindgut microbiome, respectively. Furthermore, two LPMO families (AA9 and AA10) were functionally identified as oxidoreductases corresponding to 30 modules in the microbiome, demonstrating an alternative cellulose degradation strategy present in the PBL gut microbiome. In addition, one GH family (GH94), which is known as cellobiose phosphorylase (EC 2.4.1.20), corresponding to 47 modules was functionally identified in the microbiome (Fig. 3; Additional file 8: Table S7).

Hemicellulose is a polysaccharide formed from monomeric sugars and sugar acids linked together by β-1,4- and β-1,3-glycosidic bonds [51]. Therefore, compared with that of cellulose, the degradation of hemicellulose requires a more extensive enzymatic arsenal. The current data showed that hemicellulases were the most abundant lignocellulose-degrading CAZymes in the PBL holobiont, representing 87.24%, 89.47%, and 70.27% of the identified LDMs in the midgut microbiome, hindgut microbiome, and host, respectively. Among these hemicellulase families, fifty-seven (ten CE families and 47 GH families) were identified in the microbiome, and twelve (one CE family and 11 GH families) were identified in the gut transcriptome (Fig. 2; Additional file 6: Table S6). Xylan is the main carbohydrate in hemicellulose. The functional prediction of the enzymatic activities demonstrated a multifunctional xylanolytic enzyme system present in the PBL microbiome. As shown in Fig. 3, xylan hydrolysis is involved in several enzymatic hydrolysis processes. First, the xylan backbone is randomly cleaved by endoxylanase (EC 3.2.1.8), and in the PBL microbiome, 1101 endoxylanase modules were identified, with 79.65% of them present in the hindgut microbiome. Then, the xylose polymer is broken down to its monomeric form by the action of β-xylosidase (exoxylanase, EC 3.2.1.37); 272 modules from the midgut microbiome and 1058 modules from the hindgut microbiome were predicted to have exoxylanase catalytic activity. In the PBL microbiome, various enzymes with debranching activity were also predicted, which are essential for xylan hydrolyzation [13, 52], including 137 α-glucuronidases (EC 3.2.1.139) and 460 acetylxylan esterases (EC 3.1.1.72) responsible for removal of the acetyl and phenolic side branches, 806 α-L-arabinofuranosidases (EC 3.2.1.55) catalyzing the removal of side groups, 152 feruloyl esterases (EC 3.1.1.73) cleaving the ester bonds present on xylan, and 672 α-galactosidases (EC 3.2.1.22) catalyzing hydrolysis of the terminal α-galactosyl moieties. Additionally, in softwood, mannan is the major component of hemicellulose. In the PBL gut microbiome, a total of 181 endomannosidase (EC 3.2.1.78) and 121 exomannosidase (EC 3.2.1.25) modules were also identified for mannan hemicellulose degradation. For the PBL host, only one α-galactosidase module (GH27) and one exomannosidase module (GH2) were identified in the gut transcriptome (Additional file 8: Table S7).

### Taxonomic origin of lignocellulose degradation-related genes from the PBL gut microbiome

Genes encoding lignocellulose-degrading CAZymes and lignocellulose-binding modules were searched against the NCBI NR protein database to assign taxonomic origin. In total, 12,280 genes from the midgut microbiome and 38,287 genes from the hindgut microbiome were analyzed. The results showed that 11,095 (90.35%) genes from the midgut microbiome and 33,132 (86.54%) genes from the hindgut microbiome were assigned to prokaryotic species.

When profiling the taxonomic origin, the data indicated that the PBL hindgut could selectively enrich lignocellulose-degrading microbial species. In the PBL midgut, *Firmicutes* contributed only 6.17% of lignocellulose degradation-related genes, although *Firmicutes* accounted for 52.02% of the midgut bacteria flora. However, in the hindgut, 60.46% of lignocellulose degradation-related genes were contributed by *Firmicutes*, which was higher than its relative abundance (40.55%) in the microbial community (Fig. 1A). In addition to *Firmicutes*, *Bacteroidetes* was also worth noting for its contribution of lignocellulose degradation-related genes. In the midgut, this phylum contributed 11.86% of lignocellulose degradation-related genes, although its relative abundance in the microbial community was rare (0.11%); in the hindgut, the relative abundance of *Bacteroidetes* significantly increased to 24.23%, and *Bacteroidetes* contributed 16.76% of lignocellulose degradation-related genes. At the family level, the main contributors were also concentrated in several *Firmicutes* families, including Ruminococcaceae, Lachnospiraceae, Paenibacillaceae, and Clostridiaceae, as well as one *Bacteroidetes* family, Bacteroidaceae, which contributed 57.28%, 48.16%, 52.18%, and 57.63% of cellulases, hemicellulases, CAZymes containing both cellulases and hemicellulases, and lignocellulose-binding module encoding genes, respectively (Fig. 1B). However, these families contributed very little to the lignocellulose degradation-related genes in the midgut (lower than 2.57%).

When focused on sequence novelty, our results indicated that most of the lignocellulose degradation-related genes from the PBL gut microbiome were novel. Sequence identity analysis indicated that only 5.48% of the predicted lignocellulose degradation-related proteins were highly conserved and shared more than 90% identity with the best-hit homologs in the NCBI NR database. Regarding the gut compartment, the lignocellulose-degrading enzymes and lignocellulose-binding modules enriched in the hindgut were more novel than those in the midgut. The Wilcoxon test demonstrated that the amino acid identity of LDM proteins in the hindgut was significantly lower than that in the midgut (*p* < 0.001, Fig. 1C), suggesting the potential for discovering valuable and novel enzyme resources for lignocellulose degradation from the PBL hindgut.

### Metagenomic bin reconstruction and lignocellulolytic potential

To further analyze the lignocellulolytic potential of the community at the individual microbial species level, metagenomic contig binning was performed to reconstruct the genomes from PBL gut microbial communities. In this investigation, a total of 2526 metagenomic bins were obtained (Additional file 9: Table S8), and 48.48% and 54.61% of the midgut and hindgut metagenomic reads mapped back to these bins, respectively. Completeness assessment analysis showed that 1110 bins were substantially complete (≥ 70% completeness) and that 574 bins were near complete (≥ 90%). Then, 164 substantially complete bins with low contamination levels (≤ 5%) [37] and high relative abundance were selected for subsequent analyses (Additional file 10: Fig. S1; Additional file 11: Table S9). The phylogenetic reconstruction of these 164 bins indicated that *Firmicutes* was the dominant phylum and comprised 56.10% (*N*=92) of all bins, followed by *Bacteroidetes* (*N*=21), *Actinobacteria* (*N*=13), and *Proteobacteria* (*N*=9). To evaluate the lignocellulose degradation potential of the 164 selected bins, the lignocellulose-degrading enzyme and lignocellulose-binding module of bins were analyzed. The data showed that most of these bins (*N*=156) possessed LDMs, and 71 of them were specifically notable because of their possibility of independent lignocellulose degradation, based on the possession of endo-hemicellulases, exo-hemicellulases, and debranching enzymes as well as endoglucanases and β-glucosidases.

**Fig. 4:**
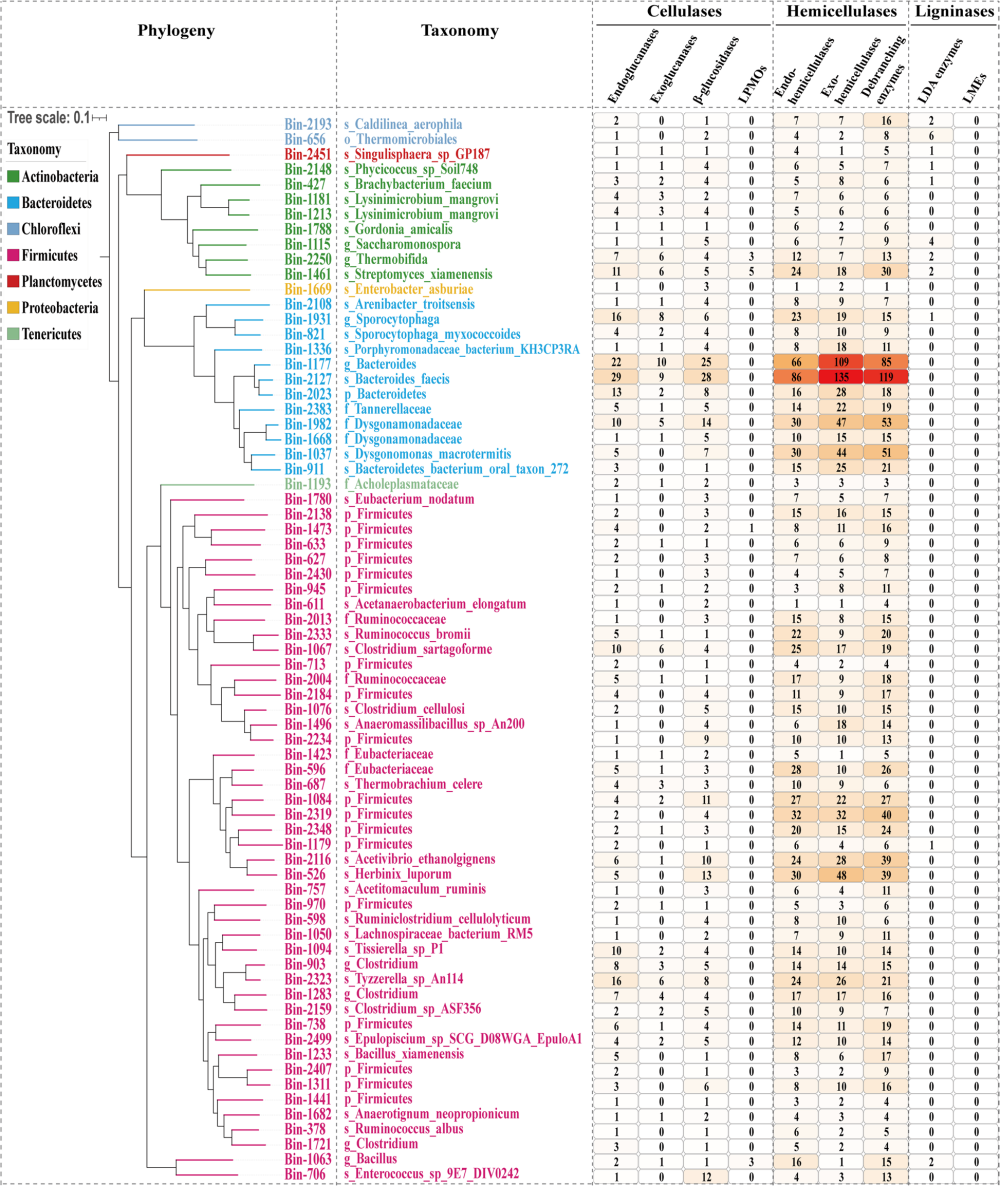
Phylogenetic affiliation, taxonomic assignment, and metabolic potential of 71 genomic bins with independent cellulose and hemicellulose degradation capability. Branches and labels with different colors represent different phyla. Taxonomic assignment level, k_, kingdom; p_, phylum; c_, class; o_, order; f_, family; g_, genus; s_, species. The heatmap in the right depicts the number of lignocellulose-degrading CAZymes in each bin

Regarding LDMs, the data showed that the distribution of exoglucanases was not universal, as these enzymes were detected in only 41 bins. In contrast, numerous endoglucanases were identified in the 71 bins, including members from the GH5, GH6, GH8, GH9, GH12, GH26, GH44, and GH51 families. These data indicated that the lack of exoglucanases may be compensated by endoglucanases [53], despite their inefficiency against crystalline cellulose. Several bins were predicted to have strong lignocellulose-degrading potential based on the composition of their LDMs. For instance, Bin-1461, Bin-2250, Bin-1063, and Bin-1473 were noteworthy for possessing LPMOs; Bin-2127 and Bin-1177 were noteworthy for possessing the largest number of cellulases and hemicellulases (Fig. 4). Furthermore, none of the 71 bins could degrade lignin due to the lack of essential LMEs for lignin degradation.

Regarding the taxonomic assignment of these bins with independent lignocellulose degradation capability, the results indicated that more than half of these bins could be novel species. Among the 71 bins, 36 bins were assigned only to the above-species level, including 19 at the phylum level, one at the order level, eight at the family level, and eight at the genus level, suggesting the presence of valuable novel microbial species resources in the PBL gut microbiota for lignocellulosic biomass conversion.

## Discussion

The highly efficient lignocellulose degradation mechanism of the larvae of the saprophagous insect PB has recently gained increasing research interest due to their potential not only in farming edible insects but also in biotechnological applications. Recently, to better promote PBL biological research and understand the genetic basis of PBL biological characteristics, we sequenced and assembled the first PB genome [25]. The subsequent gene annotation showed that PBL are not able to complete the process of lignocellulose degradation by themselves, indicating that the highly efficient lignocellulose degradation in PBL may be attributed to their microbial symbionts. In this work, we investigated for the first time the complete enzyme repertoire for lignocellulose degradation on the scale of holobiont in PBL. Combining the host gut transcriptome with the gut metagenomic data, we established a complete gut reference gene catalog that allowed us to further characterize both endogenous and microbial enzymes associated with the breakdown of lignocellulose in the PBL gut.

**Fig. 5:**
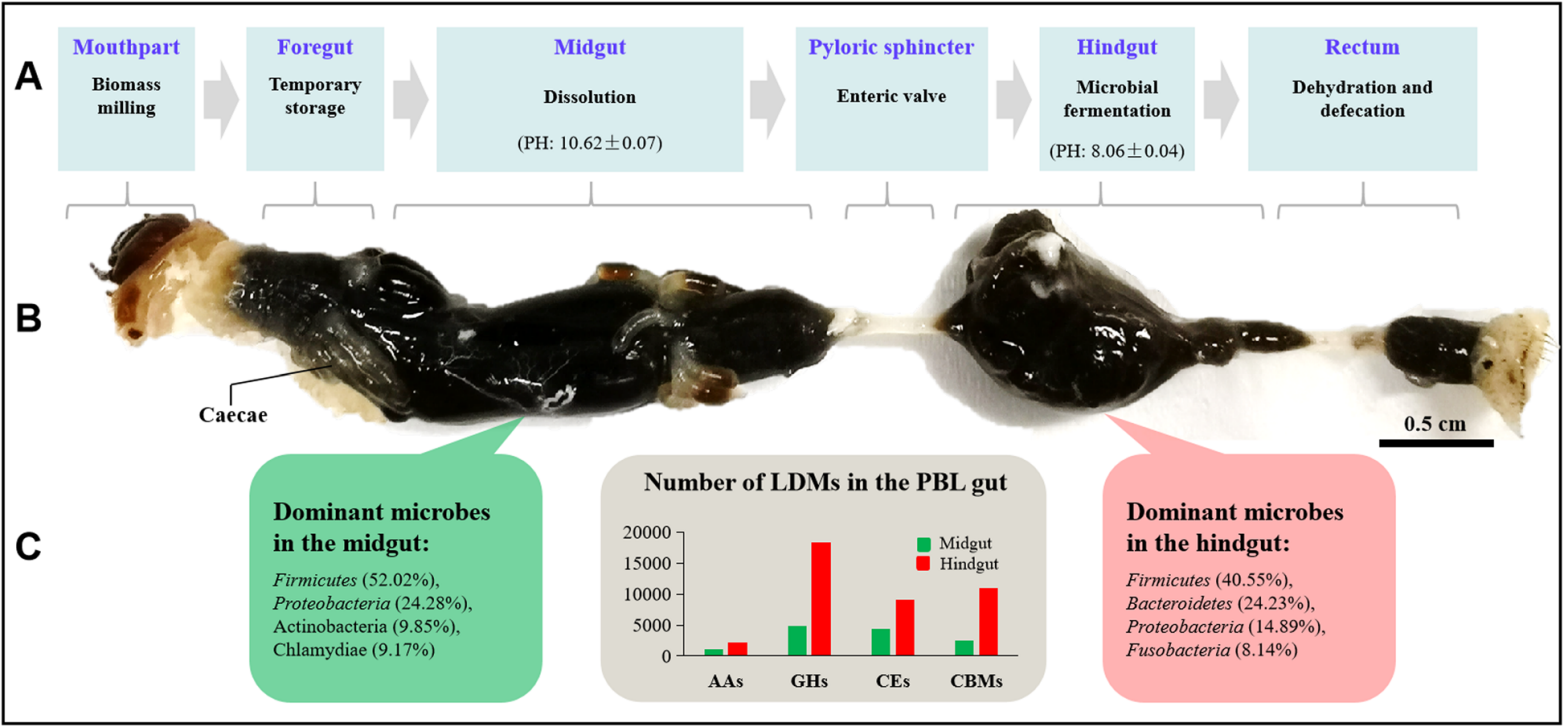
Structural and functional assembly the of PBL digestive tract. Physicochemical properties and major functions in the lignocellulose conversion of different digestive tract compartments (**A**). The view of the digestive tract of PBL showing relative locations of different compartments (**B**). Dominant microbes and the total number of LDMs in the PBL midgut and hindgut microbiome (**C**)

Overall, the PBL holobiont was assembled like a mini automatic production line for lignocellulose degradation (Fig. 5). First, PBL feeding habits drive the production line. In nature, scarab larvae such as PBL are attracted to carbon dioxide (CO_2_) [54], which drives saprophagous PBL to feed on decaying organic matter. This feeding is beneficial to many aspects of lignocellulose degradation in the PBL holobiont: (a) the PBL chews and crushes lignocellulosic biomass, which may reduce recalcitrance of the substrate and allow PBL to achieve greater lignocellulose degradation efficiency [55]; (b) the PBL ingests a large number of lignocellulose-decomposing bacteria from the decaying organic matters, most of which will be killed and hydrolyzed in the midgut, providing nutrients for PBL development [56], while some surviving from the midgut will contribute to the hindgut lignocellulosic bacteria flora [57]. Beyond feeding habits, a strong alkaline environment in the PBL midgut facilitates the solubility of organic polymers. Organic carbon analysis of intestinal contents showed that more than half of the biomass was solubilized in the highly alkaline midgut (Additional file 12: Table S10), which was capable of rendering organic components accessible to enzymatic digestion [8]. The hindgut of scarab larvae is considered to be the primary site for lignocellulose digestion, analogous to the rumen of herbivore ruminants and known as the “fermentation chamber” [58, 59]. In the present study, the annotation of metagenomic data showed a high abundance and diversity of microbial lignocellulose degradation-related enzymes in the PBL hindgut. Then, the organic matter fermented in the hindgut is dehydrated in the rectum, forming granular feces, and excreted. Therefore, the unique feeding and digestion process of PBL demonstrates that the PBL holobiont can be used as a valuable research model to study the degradation and utilization of lignocellulose.

The PBL reference gene catalog and gut content analysis data illustrate a unique teamwork between the PBL and its gut bacterial flora. In some holobionts, lignocellulose degradation is achieved via teamwork by the host and symbionts, such as in wood-feeding termites [60, 61] and omnivorous-feeding pill bugs [62], in which the lignocellulose degradation enzymatic cocktail is complemented by enzymes produced by both the host and the symbionts. However, in the PBL holobiont, the symbiont plays the major role in lignocellulose degradation processes by providing a complete and abundant enzyme repertoire. The host provides only a very limited number of enzymes involved in the depolymerization of lignocelluloses, but it poses a functional complement by providing lignin pretreatment processes. Pretreatment is the first step in the lignocellulosic material utilization process to promote access to cellulose and hemicellulose, including various physical or chemical pretreatment approaches [63]. Among these, physical pretreatment, such as grinding or milling, can reduce the particle size of materials, and chemical alkali pretreatment can lead to the separation of structural linkages between lignin and carbohydrates and disruption of the lignin structure, which have been widely applied in the pulp industry [64, 65]. In our model, the PBL host was able to provide both physical and chemical pretreatment options by evolving strong mouthparts as well as a strong alkaline environment in the midgut, which could complement the lack of laccases [66] in the PBL holobiont and promote the subsequent enzymatic hydrolysis of cellulose and hemicellulose in the hindgut. The analysis of the consumption of organic carbon during the PBL conversion process confirmed the functional complementation of the PBL host (Additional file 12: Table S10).

Regarding the enzymes, a broad array of cellulases, hemicellulases, and ligninases for lignocellulosic biomass degradation were identified in the hindgut microbiome, which were similar to that of cow rumen metagenome both in enzyme numbers and diversity [58]. Although laccase, an important lignin-degrading enzyme [66] was lacking in both the PBL hindgut and rumen metagenomes, the number of peroxidases and LDA enzymes in the PBL hindgut were higher than that in the rumens of cow [58] and camel [59] (Additional file 13: Table S11), demonstrating the capability of lignin modification in the PBL hindgut microbiome. Microbial source analysis demonstrated that these LDM-encoding genes in the PBL hindgut were similar to those in rumens and were mainly contributed by two phyla, *Firmicutes* and *Bacteroidetes*, although with different levels of contribution [59, 67]. Members of these two phyla are known as potent lignocellulose degraders, and their association with lignocellulose degradation has been well established [68]. Furthermore, our data indicated that most of the LDM sequences enriched by the PBL symbiont were novel, sharing lower than 90% identity with the best-hit homologs in the NCBI NR database, similar to cow rumen [58]. These data point to valuable and novel genetic resources for carbohydrate degradation in the PBL microbiome.

Regarding the microbiota, further taxonomic assignment at the individual microbial species level proved the existence of diverse and novel lignocellulosic microflora present in the PBL gut. A total of 156 high-quality metagenomic bins showing lignocellulolytic potential were reconstructed in this work, and as expected, 70% of these bins were associated with species belonging to *Firmicutes* and *Bacteroidetes*. Among these, 71 bins were identified with independent lignocellulose degradation capability and most (50.70%) of these genomes represent previously unsequenced strains and species, demonstrating discovery of novel bacterial species associated with lignocellulose degradation in the PBL microbiota. To assess the reliability of the metagenome binning results in this work, for 35 bins resolvable to the species level, we collected the public genomes of the same species from the NCBI database and analyzed the potential of lignocellulose degradation for these published genomes. The data indicated that the LDM genes were also present in all these published genomes (Additional file 14: Table S12). Some of the species these bins assigned to were isolated from known lignocellulolytic organisms, such as ruminants or termites, and have been proven to have lignocellulose degradation capability. For instance, Bin-2127 was identified as a strain of *Bacteroides faecis*, a species isolated from human feces and confirmed to be a decomposer of various mono/polysaccharides [69]. Bin-2127 possessed the highest number (273) of LDMs, which was similar to the 189 LDM genes in the published *B. faecis* strain genome (GCA_000226135.1). Another *Bacteroidetes* bin (Bin-821) was assigned to *Sporocytophaga myxococcoides*, a species that has been regarded as a highly efficient carbohydrate metabolizer possessing a wide array of cellulolytic enzymes [70]. The annotation of its public genome (GCA_000426725.1) revealed a complete set of endoglucanases, exoglucanases, and β-glucosidases for cellulose degradation. Among *Firmicutes* species, Bin-1076 was assigned to *Clostridium cellulosi*, Bin-1067 to *Clostridium sartagoforme*, Bin-526 to *Herbinix luporum*, and Bin-2333 to *Ruminococcus bromii*, which are also capable of utilizing various carbon sources, including inulin, mannitol, sucrose, crystalline cellulose, or plant polysaccharides [71–74]. These bins and corresponding public genomes also possessed a high number of cellulase and hemicellulase genes (Additional file 14: Table S12). Overall, the consistency of LDM genes between the reconstructed bins and the corresponding species’ genomes further illustrated the representativeness of the PBL metagenomic features we demonstrated in this study, as well as the research value and application prospects of the PBL lignocellulose degradation model.

## Conclusions

In summary, a comprehensive reference catalog of gut microbial genes and host gut transcriptomic genes was first established in this work for PBL at the holobiont level. The investigation not only elucidates the microbial species that contribute to lignocellulose degradation but also reveals a new association between host and symbiotic microorganisms in the PBL holobiont. The traditional hologenome theory mainly focuses on the genetic wealth of diverse microbial symbionts and suggests that they can play an important role both in the adaptation and evolution of hosts. However, in the PBL holobiont, we illustrated that the host’s organic functional complementation may play a more durable and stable role for the holobiont in lignocellulose degradation and may facilitate its survival and multiplication in the ecosystem. Therefore, this discovery will expand the knowledge of holobionts and open a new beginning in the theory of holobionts.

## Supplementary Information

**Additional file 1: Table S1**. Information on the sampling and sequencing of PBL transcriptome and metagenome.

**Additional file 2: Table S2**. FPKM values for 8,505 genes identified in the midgut and hindgut transcriptomes of PBL.

**Additional file 3: Table S3**. Taxonomic annotation of the PBL gut microbial community. The percentage of estimated taxa from the phylum level to the species level in the community are shown for midgut and hindgut microbiome respectively.

**Additional file 4: Table S4**. The total numbers of CAZy modules for each family identified in the PBL host and in the gut microbiome.

**Additional file 5: Table S5**. The average expression levels (mean and SD) for CAZy modules represented in the midgut and hindgut transcriptomes of PBL.

**Additional file 6: Table S6**. Lignocellulose-degrading CAZymes and lignocellulose-binding modules identified in the PBL host and in the gut microbiome.

**Additional file 7.** Protein sequences of identified lignocellulose-degrading CAZymes genes in the PBL gut transcriptome and metagenome.

**Additional file 8: Table S7**. The predicted enzymatic function of CAZy modules identified in the PBL host and in the gut microbiome.

**Additional file 9: Table S8**. Basic genome characteristics of 2,526 recovered genomic bins.

**Additional file 10: Figure S1**. Phylogenetic affiliation, relative abundance and metabolic potential of 164 genomic bins from the PBL microbiota. The phylogenetic tree and the taxonomic assignment of reconstructed bins are shown as the innermost layers. Branches and labels with different colors represent different phyla. Labels with pink background represent 71 bins with independent (hemi) cellulose degradation capability. Bootstrap values over 0.9 are indicated using filled purple circles on the branch. The heatmap in the third layer depicts the relative abundance of the 164 bins in the midgut and hindgut metagenomic samples respectively. The heatmap in the outermost four layers depicts the number of CAZy modules involved in lignocellulose degradation in each bin.

**Additional file 11: Table S9**. Basic genome characteristics and lignocellulolytic potential of 164 recovered genomic bins.

**Additional file 12: Table S10**. Organic carbon analysis of intestinal contents in the PBL midgut and hindgut.

**Additional file 13: Table S11**. Counts of lignocellulose degradation-related modules in the PBL hindgut, cow and camel rumen microbiomes. The CAZyme profiles of PBL hindgut microbiome were compared with those present in cow and camel rumens, using same methods as in study of Gharechahi et al (2018). Contigs longer than 1000 nt were subjected to ORF prediction and a dbCAN database search.

**Additional file 14: Table S12**. Comparison of lignocellulose degradation-related modules in the bins recovered in this work and corresponding public species genomes from the NCBI database.

## Data Availability

The datasets generated and/or analyzed during the current study are available in the NCBI SRA repository under accession numbers provided in Additional file 1.
