# Supplementary figures and images for: Lignocellulose degradation in Protaetia brevitarsis larvae digestive tract: refining on a tightly designed microbial fermentation production line
Source: Microbiome. 2022 Jun 13;10:90. doi: 10.1186/s40168-022-01291-2 (PMC9195238; doi:10.1186/s40168-022-01291-2)

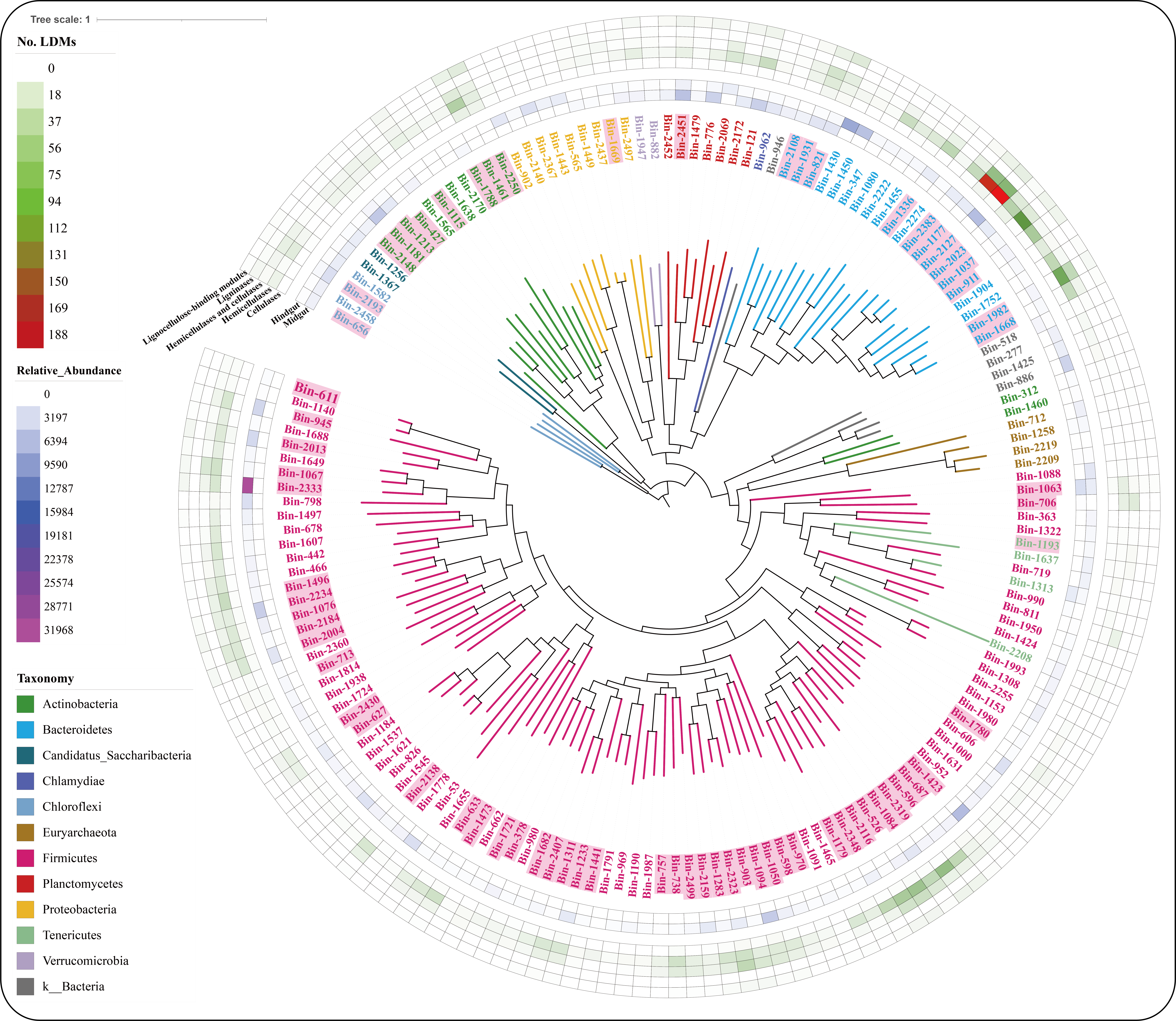

Supplement: Supplementary file 11 — Additional file 10: Figure S1. Phylogenetic affiliation, relative abundance and metabolic potential of 164 genomic bins from the PBL microbiota. The phylogenetic tree and the taxonomic assignment of reconstructed bins are shown as the innermost layers. Branches and labels with different colors represent different phyla. Labels with pink background represent 71 bins with independent (hemi) cellulose degradation capability. Bootstrap values over 0.9 are indicated using filled purple circles on the branch. The heatmap in the third layer depicts the relative abundance of the 164 bins in the midgut and hindgut metagenomic samples respectively. The heatmap in the outermost four layers depicts the number of CAZy modules involved in lignocellulose degradation in each bin. [file 40168_2022_1291_MOESM10_ESM.png]
